# Effect of micro-aeration on syntrophic and methanogenic activity in anaerobic sludge

**DOI:** 10.1007/s00253-023-12969-4

**Published:** 2024-02-02

**Authors:** Bruno P. Morais, Carla P. Magalhães, Gilberto Martins, Maria Alcina Pereira, Ana J. Cavaleiro

**Affiliations:** 1 CEB - Centre of Biological Engineering, University of Minho, Braga, Portugal; 2 LABBELS – Associate Laboratory, Braga/Guimarães, Portugal; 3 Present Address: CICECO - Aveiro Institute of Materials, Universidade de Aveiro, Aveiro, Portugal

**Keywords:** Micro-aeration, Facultative anaerobic bacteria, Syntrophy, Methane

## Abstract

**Abstract:**

Micro-aeration was shown to improve anaerobic digestion (AD) processes, although oxygen is known to inhibit obligate anaerobes, such as syntrophic communities of bacteria and methanogens. The effect of micro-aeration on the activity and microbial interaction in syntrophic communities, as well as on the potential establishment of synergetic relationships with facultative anaerobic bacteria (FAB) or aerobic bacteria (AB), was investigated. Anaerobic sludge was incubated with ethanol and increasing oxygen concentrations (0–5% in the headspace). Assays with acetate or H_2_/CO_2_ (direct substrates for methanogens) were also performed. When compared with the controls (0% O_2_), oxygen significantly decreased substrate consumption and initial methane production rate (MPR) from acetate or H_2_/CO_2_. At 0.5% O_2_, MPR from these substrates was inhibited 30–40%, and close to 100% at 5% O_2_. With ethanol, significant inhibition (>36%) was only observed for oxygen concentrations higher than 2.5%. Oxygen was consumed in the assays, pointing to the stimulation of AB/FAB by ethanol, which helped to protect the syntrophic consortia under micro-aerobic conditions. This highlights the importance of AB/FAB in maintaining functional and resilient syntrophic communities, which is relevant for real AD systems (in which vestigial O_2_ amounts are frequently present), as well as for AD systems using micro-aeration as a process strategy.

**Key points:**

•*Micro-aeration impacts syntrophic communities of bacteria and methanogens.*

•*Oxygen stimulates AB/FAB, maintaining functional and resilient consortia.*

•*Micro-aeration studies are critical for systems using micro-aeration as a process strategy.*

**Supplementary Information:**

The online version contains supplementary material available at 10.1007/s00253-023-12969-4.

## Introduction

The current geopolitical situation imposes an acceleration in the quest for clean energy and green transition. Intensification of biomethane production is one of the short-term measures proposed by the European Commission to attain this objective (European Commission 2022). In particular, biomethane production from organic waste/wastewater through anaerobic digestion (AD) will have a role in the reduction of fossil fuel consumption and in the decarbonization of the energy system (European Biogas Association 2022).

In recent years, micro-aeration has been pointed out as an attractive strategy to improve AD processes (Botheju and Bakke 2011; Fu et al. 2023; Nguyen and Khanal 2018). Beneficial effects of injecting limited amounts of oxygen have been reported, namely scavenging H_2_S, enhancing the hydrolysis step, and avoiding volatile fatty acids (VFA) accumulation, thereby improving the overall stability of AD systems (Nguyen et al. 2019b; Nguyen and Khanal 2018; Tsapekos et al. 2017; Xu et al. 2014). These positive effects have been related to the activity of facultative anaerobic bacteria (FAB) since micro-aeration creates a unique environment that enables both anaerobic and micro-aerobic activities to occur within a single reactor (Nguyen and Khanal 2018). FAB have been frequently detected in several works and associated with the positive effects reported (Cavaleiro et al. 2016; Duarte et al. 2020; Nguyen et al. 2019a).

Besides FAB, the conversion of organic matter to methane in AD systems relies on the coordinated activity of different microbial groups. In particular, syntrophy has an essential role in the anaerobic breakdown of organic compounds in methanogenic ecosystems (McInerney et al. 2009). Syntrophy is a tightly coupled mutualistic interaction, where hydrogen/formate is exchanged between the partners and must be kept at low concentrations, for efficient cooperation among the partners to occur (Sieber et al. 2012). Methanogens and other strict anaerobes are active in environments with low redox potential (Jasso-Chávez et al. 2015). As such, exposure to oxygen can potentially disturb and hamper these microorganisms’ growth.

Most studies regarding the potentially toxic effects of oxygen in AD have focused on methanogens in pure/co-cultures or mixed cultures (sludge). Exposure to oxygen was shown to be detrimental to methanogens, due to the formation of reactive oxygen species (ROS) such as superoxide anions (^●^O_2_^−^) and hydrogen peroxide (H_2_O_2_). Previously, it was believed that these microorganisms lacked the mechanisms to cope with oxidative stress, but several studies have shown that methanogens can survive oxygen exposure for hours or days (Fetzer and Conrad 1993; Jasso-Chávez et al. 2015; Kiener and Leisinger 1983; Patel et al. 1984). Moreover, active methanogenic communities have been found in typical oxidative environments (Angle et al. 2017; Yasin et al. 2015).

Antioxidative defense mechanisms have been found in some methanogenic archaeal species, that help them cope with excessive intercellular ROS and to alleviate oxidative stress. These mechanisms are mainly associated with specific ROS scavenging enzymes, such as superoxide dismutase (SOD), anaerobe-specific superoxide reductase (SOR), catalase, and F_420_H_2_ oxidase (FprA) (Li et al. 2022). Lyu and Lu (2018) reported the occurrence of a systematic shift in metabolism across members of the two classes of methanogens (class I, containing *Methanococcales*, *Methanopyrales*, and *Methanobacteriales* and class II, containing *Methanomicrobiales*, *Methanocellales*, and *Methanosarcinales* (Brochier-Armanet et al. 2011; Lyu and Lu 2018): Class II methanogens possess expanded antioxidant features that enable better oxidative adaptation and are more frequently recovered from micro-aerobic and oxic environments, than Class I methanogens.

An in-depth analysis of the effects of oxygen on strict anaerobes is still necessary, for the further development of large-scale micro-aeration processes and for the control and optimization of most AD treatment systems. These are generally not operated under strict anaerobic conditions, and vestigial oxygen amounts are most frequently present. In particular, the effect of low oxygen concentrations on the activity and interaction between syntrophic bacteria and methanogens, as well as on the potential establishment of synergetic relationships between these microorganisms with FAB, is far from being fully understood. In the present work, the effect of low oxygen concentrations (up to 5%) on the activity of a syntrophic methanogenic community was studied, using anaerobic sludge as inoculum and ethanol as substrate.

## Materials and methods

### Micro-aerobic assays

Assays were performed in triplicate, in 160 mL serum bottles with 55 mL working volume. A bicarbonate-buffered mineral salt medium was prepared as described by Stams et al. (1993). No reducing agent was added, and therefore additional measures were adopted to minimize O_2_ diffusion to the medium in each step. Anaerobic granular sludge was collected from a brewery wastewater treatment plant (Super Bock, Leça do Balio, Portugal) and used as inoculum. The specific methanogenic activity (SMA) of the inoculum was determined according to Pereira et al. (2005) and expressed in mL of methane at standard temperature and pressure (STP) conditions per amount of inoculum (g of volatile solids, VS) and per day. In the presence of acetate (30 mmol L^−1^), ethanol (30 mmol L^−1^), or H_2_/CO_2_ (80/20% v/v, P = 1.7 × 10^5^ Pa), SMA values were 24 ± 1 mL g^−1^ d^−1^, 671 ± 60 mL g^−1^ d^−1^, and 878 ± 79 mL g^−1^ d^−1^, respectively.

In the assays, the sludge was disrupted and added to the bottles at a final VS concentration of 4 g L^−1^. The bottles were closed with butyl rubber stoppers and aluminum crimp caps and were flushed with N_2_/CO_2_ (80:20% v/v), at a final pressure of 1.7 × 10^5^ Pa. Ethanol (30 mmol L^−1^) was added as substrate. In parallel, assays with acetate (30 mmol L^−1^) or H_2_/CO_2_ (80/20% v/v, 1.7 × 10^5^ Pa), which are direct substrates for methanogens, were also prepared, as well as blank assays (receiving no substrate).

The experiment comprised two distinct phases. In phase one (P1), the cultures were incubated under anaerobic conditions until the substrate added was totally consumed, except in the case of acetate which was only half consumed (its degradation was slower). Then, in phase two (P2), oxygen was added to the bottles. For that, the headspace of the bottle was flushed with N_2_/CO_2_ (assays amended with acetate and ethanol) or H_2_/CO_2_, followed by air injection using an N_2_-flushed glass gas-tight syringe (SGE Analytical Science, Trajan, Ringwood, Victoria, Australia), with final pressure adjusted to 1.0 × 10^5^ Pa (1 atm) in all bottles. Increasing O_2_ concentrations were applied in the bottles’ headspace (i.e., 0%, 0.5%, 1%, 2.5%, and 5%). Bottles were again supplemented with the respective substrates, at the same concentration for ethanol and H_2_/CO_2_, or half for acetate. The transition from P1 to P2 was defined based on the cumulative methane production values and on the stoichiometry of the expected reactions (Table 1).

**Table 1:** Stoichiometry of the reactions involved in syntrophic ethanol degradation to CH_4_

Reaction	Equation	Δ*G*°′ (kJ reaction^−1^) ^(a)^
1. Ethanol oxidation to acetate and H_2_	CH_3_CH_2_OH + H_2_O → CH_3_COO^−^ + H^+^ + 2 H_2_	9.6^(b)^
2. Methane production from acetate	CH_3_COO^−^ + H^+^ → CH_4_ + CO_2_	−36^(c)^
3. Methane production from H_2_/CO_2_	4 H_2_ + CO_2_ → CH_4_ + 2 H_2_O	−131^(c)^
4. Syntrophic ethanol oxidation to acetate and methane	2 CH_3_CH_2_OH + CO_2_ → 2 CH_3_COO^−^ + 2 H^+^ + CH_4_	−111.8
5. Total ethanol oxidation to methane	2 CH_3_CH_2_OH → 3 CH_4_ + CO_2_	−183.8

^(a)^Δ*G*°′ (Gibbs free energy change at standard conditions, i.e., solute concentrations of 1 mol L^−1^, gas partial pressure of 10^5^ Pa, T = 25 °C, pH 7). ^(b)^(Thauer et al. 1977). ^(c)^(Stams and Plugge 2009)

All cultures were incubated at 37 °C and 110 rpm, in the dark. Methane was measured over time. Oxygen, hydrogen, acetate, and ethanol were periodically measured.

### Analytical methods

Gas samples (0.5 mL) were collected from the bottles’ headspace using a glass gas-tight syringe (Trajan Scientific, Australia). For CH_4_ quantification, a Shimadzu GC-2014 (Shimadzu; Japan) was used, equipped with a Porapack Q (100–180 mesh) column and a flame ionizing detector (FID), with N_2_ as carrier gas at a 30 mL min^−1^ flow. Temperatures of the injection port, column, and detector were 110 °C, 35 °C, and 220 °C, respectively. A mixture of CH_4_/CO_2_/N_2_ (40:40:20% v/v) was used as standard. For H_2_ and O_2_ quantification, a Bruker SCION GC-486 (Billerica, MA, USA) was used, equipped with a Molsieve packed column (13 × 80/100, 2 m length, 2.1 mm internal diameter) and a thermal conductivity detector (TCD), with argon as the carrier gas at 30 mL min^−1^. Temperatures of the injector, column, and detector were 100 °C, 35 °C, and 130 °C, respectively. Mixtures of H_2_/CO_2_ (80:20% v/v) and air (21% O_2_ v/v) were used as standards for H_2_ and O_2_ quantification, respectively. For acetate and ethanol analysis, samples were centrifuged at 15,000 rpm for 10 min, after which the supernatant was collected and filtered with a 0.22 μm filter. High-performance liquid chromatography (HPLC) was performed in a liquid chromatograph (Jasco, Tokyo, Japan) equipped with an Aminex 87H column (300 × 7.7, 8 μm particle size-Bio-Rad, CA, USA) at 60 °C, and a Jasco UV-2075 Plus (*λ* = 210 nm) and a Jasco RI-4030 detectors, for acetate and ethanol, respectively. Each sample was run at 0.7 mL min^−1^ using a 5 mmol L^−1^ H_2_SO_4_ (HPLC grade) solution as the mobile phase. Crotonic acid was used as an internal standard at a 4:1 (sample/crotonic ac.) volume ratio.

### Calculation of the inhibitory effect of oxygen and statistical analysis

Methane production rate (MPR) was determined by calculating the initial slope of the cumulative methane production curves in P1 and P2. The ratio between the MPR in P2 and P1—slope ratio (Sr)—was calculated for each incubation condition, to correct for changes observed upon phase transition in the controls (Silva et al. 2016). Slope ratio values calculated for increasing O_2_ concentrations were then compared to the ones from the controls (0% O_2_), and the inhibitory effect (%) of O_2_ was calculated (Eq. 1).

1 $$Inhibition\ \left(\%\right)=\frac{Sr_{control}-{Sr}_{O2}}{Sr_{control}}\times 100$$

where

- Sr_control_ = Sr obtained in the control assays (0% O_2_)
- Sr_O2_ = Sr obtained in the assays supplemented with O_2_.

The statistical significance of the differences observed in the results achieved was evaluated using single-factor analysis of variances (ANOVA). An *F*-test was applied between pairs of data (comparison between a control set and a treated set) to evaluate the equality of variances to determine the most appropriate statistical *T*-test. Statistical significance was established at the *p* < 0.05 level.

## Results

**Fig. 1:**
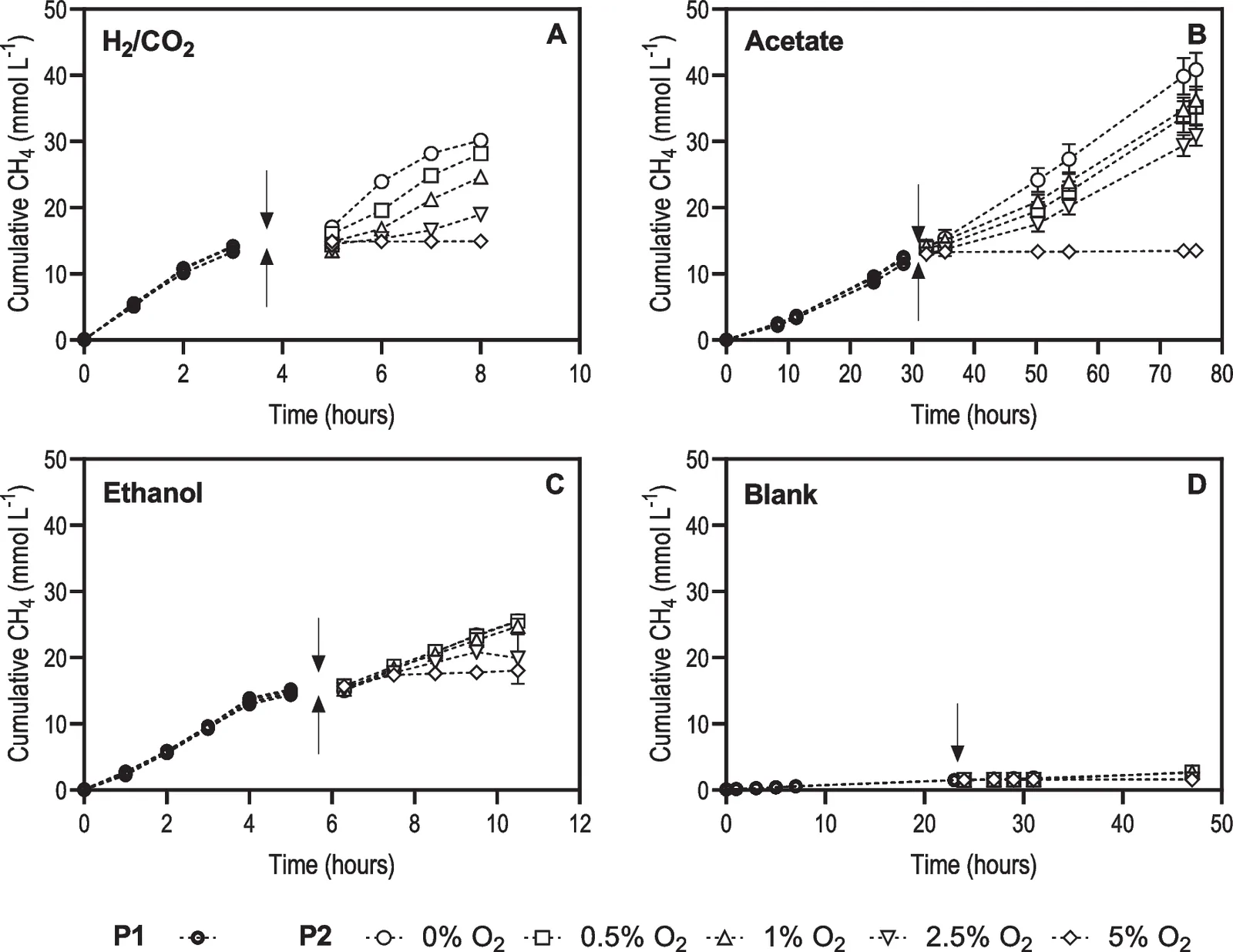
Effect of different O_2_ concentrations on cumulative methane production from H_2_/CO_2_ (**A**), acetate (**B**), and ethanol (**C**). Cumulative methane production in the blanks is also shown (**D**). P1, before O_2_ addition (darker circles). In P2: 0% O_2_ (white circle), 0.5% O_2_ (white square), 1% O_2_ (white triangle), 2.5% O_2_ white triangle), and 5% O_2_ (white diamond). The arrow going down indicates the moment of air addition and the arrow going up indicates substrate replenishment. Each data point represents the average of triplicates ± standard deviation

For each of the substrates tested, maximum cumulative methane production and MPR were similar in all the bottles during P1 (in the absence of oxygen) (Fig. 1, Table 2, Figures S1–S3). For H_2_/CO_2_, maximum cumulative methane production reached the expected stoichiometric value in 4 h (Fig. 1A), while approximately 30 h of incubation was necessary for the bioconversion to methane of half of the initially added acetate (30 mmol L^−1^) (Fig. 1B, Table S1), due to the low aceticlastic methanogenic activity of the inoculum. As such, in P1, MPR was higher in the assays with H_2_/CO_2_ than in the assays with acetate (Table 2, Figures S1–S2), which agreed with the SMA tests.

**Table 2:** Methane production rate (MPR) from H_2_/CO_2_, acetate, and ethanol, in P1 and P2, at increasing O_2_ concentrations, slope ratio (Sr), and inhibition percentage. MPR calculated for the blank assays is also shown. Values are the average of triplicates ± standard deviation

Substrate	P1	P2		Sr	Inhibition (%)
	MPR (mmol L^−1^ h^−1^)	O_2_ (%)	MPR (mmol L^−1^ h^−1^)		
H_2_/CO_2_	5.32 ± 0.12	0	5.66 ± 0.09	1.07 ± 0.03	-
	5.41 ± 0.31	0.5	*3.98 ± 0.03	0.74 ± 0.04	31 ± 5
	5.03 ± 0.07	1.0	*3.18 ± 0.05	0.63 ± 0.01	41 ± 3
	5.03 ± 0.05	2.5	*1.14 ± 0.08	0.23 ± 0.02	79 ± 4
	5.31 ± 0.12	5.0	*0.02 ± 0.01	0.03 ± 0.00	98 ± 4
Acetate	0.48 ± 0.02	0	0.58 ± 0.04	1.22 ± 0.09	-
	0.49 ± 0.03	0.5	*0.36 ± 0.02	0.74 ± 0.06	39 ± 10
	0.50 ± 0.02	1.0	*0.42 ± 0.02	0.83 ± 0.05	31 ± 9
	0.46 ± 0.03	2.5	*0.30 ± 0.00	0.65 ± 0.04	46 ± 9
	0.46 ± 0.02	5.0	*0.01 ± 0.00	0.01 ± 0.00	99 ± 11
Ethanol	3.39 ± 0.08	0	2.52 ± 0.12	0.74 ± 0.04	-
	3.65 ± 0.14	0.5	2.30 ± 0.08	0.63 ± 0.03	15 ± 7
	3.67 ± 0.20	1.0	2.22 ± 0.11	0.60 ± 0.04	19 ± 8
	3.65 ± 0.15	2.5	*1.73 ± 0.06	0.47 ± 0.03	36 ± 7
	3.80 ± 0.09	5.0	*0.22 ± 0.05	0.06 ± 0.01	92 ± 8
Blank	0.06 ± 0.00	0	0.04 ± 0.00	-	-
	0.06 ± 0.00	0.5	0.02 ± 0.00	-	-
	0.06 ± 0.00	1.0	0.01 ± 0.00	-	-
	0.06 ± 0.00	2.5	0.00 ± 0.00	-	-
	0.06 ± 0.00	5.0	0.00 ± 0.00	-	-

*Statistically significant differences with *p* < 0.05, compared to the corresponding control assays (0% O_2_)

In the assays with ethanol, this substrate was completely consumed in P1 (Table S2), leading to a cumulative methane production of approximately 15 mmol L^−1^ in less than 6 h (Fig. 1C). This value closely matches the methane production that can be expected from the hydrogen generated from syntrophic ethanol oxidation (reactions 1 and 4—Table 1). In fact, close to stoichiometric acetate concentrations accumulated in the medium (Table 1, Table S2), showing that the methane measured during P1 (Fig. 1C) results mainly from hydrogenotrophic methanogenesis.

In the blanks (without any added substrate), the calculated MPR was substantially lower than in the other tested conditions (Fig. 1D, Table 2). With no substrate available, methane production tends to be very low resulting mainly from the consumption of residual substrate or endogenous respiration. In this case, the contribution of the background methane production can thus be considered negligible.

Changes observed in the MPR of each control (0% O_2_), in P2 relatively to P1, are most likely present in all the other conditions tested. This was the reasoning behind the calculation of the Sr (ratio between the MPR in P2 and P1) to compare each assay with the control and calculate the inhibition percentage. Upon transition to P2, MPR in the controls increased relatively to P1 in the assays with H_2_/CO_2_ and acetate, possibly due to culture acclimation or biomass growth (Fig. 1A and 1B, Table 2, Figures S1–S2). However, it decreased in the assays with ethanol (Fig. 1C, Table 2, Figure S3), which may be related with the acetate accumulation in the medium (Table S2).

During P2, oxygen exposure significantly decreased the total substrate consumption (*p* < 0.05), as well as the MPR (*p* < 0.05) relatively to the controls, in the assays with H_2_/CO_2_ or acetate, at all the O_2_ concentrations tested (Table S1, Fig. 1A and 1B, Table 2). However, in the assays with ethanol, a significant effect (*p* < 0.05) on these parameters, as well as on acetate production from ethanol, was observed only at 2.5% and 5% O_2_ (Table 3, Figure S4, Table S2, Fig. 1C, Table 2). For example, MPR from H_2_/CO_2_ or acetate was inhibited by 31 ± 5% and 39 ± 10%, respectively, at 0.5% O_2_, while similar MPR inhibition (36 ± 7%) was only observed at 2.5% O_2_ in the assays with ethanol (Table 2). At the end of P2, H_2_ was detected in the headspace of the bottles at concentrations around 0.15 mmol L^−1^ in the assays with 2.5% and 5% O_2_, being lower than that in the other assays (data not shown).

**Table 3:** Ethanol consumption and acetate production during P2, in the assays with ethanol, at increasing O_2_ concentrations. Values are the average of triplicates ± standard deviation

O_2_ (%)	Ethanol consumption			Acetate production	
	Total (%)^(a)^	Rate (mmol L^−1^ h^−1^)	Inhibition (%)^(b)^	Total (%)^(c)^	Rate (mmol L^−1^ h^−1^)
0	86 ± 8	4.3 ± 0.3	-	92 ± 9	4.0 ± 0.3
0.5	80 ± 4	4.1 ± 0.1	7 ± 1	97 ± 10	3.9 ± 0.2
1.0	79 ± 3	4.1 ± 0.4	7 ± 1	97 ± 5	3.7 ± 0.2
2.5	74 ± 7	*3.2 ± 0.2	20 ± 1	92 ± 10	*2.9 ± 0.1
5.0	25 ± 3	*0.9 ± 0.1	79 ± 6	109 ± 18	*1.1 ± 0.1

^(a)^Total ethanol consumption (%) = ([Eth]_t0_-[Eth]_tf_) × 100/[Eth]_t0_) (values in Table S2). ^(b)^Inhibition of the ethanol consumption rate, calculated by comparison to the control. ^(c)^Total acetate production (%) = ([Ac]_tf_-[Ac]_t0_) × 100/[Eth]_consumed_) (values in Table S2), considering that 1 mol of acetate is formed per mol of ethanol consumed (reaction 1—Table 1). *Statistical significance with *p* < 0.05, compared to the controls

Oxygen concentration in the headspace was also measured at the beginning and end of P2 in the assays with ethanol (Table 4), showing that most of the O_2_ present in the headspace had been consumed at the end of the experiment. However, relatively higher values were still present in the assays that received 2.5% and 5% O_2_ (i.e., 0.14 and 0.40 mmol L^−1^, respectively—Table 4, this last value corresponding to approx. 1% O_2_ in the headspace). This indicates that, in these two conditions, the cultures were exposed to O_2_ throughout the entire assay, while at 0.5% and 1%, O_2_ was readily consumed.

**Table 4:** Oxygen concentration in the headspace at the beginning (t_0_) and end (t_f_) of P2, in the assays with ethanol. Values are the average of triplicates ± standard deviation

Theoretical O_2_ concentration added		O_2_ concentration measured (mmol L^−1^)^(a)^	
(%)	mmol L^−1(a)^	*t*_0_	*t*_f_
0	0	0.05 ± 0.00	0.04 ± 0.01
0.5	0.2	0.23 ± 0.02	0.06 ± 0.00
1.0	0.4	0.41 ± 0.03	0.07 ± 0.02
2.5	1.0	1.02 ± 0.05	0.14 ± 0.02
5.0	2.0	1.93 ± 0.04	0.40 ± 0.09

^(a)^Expressed per unit volume of bottles’ headspace

**Fig. 2:**
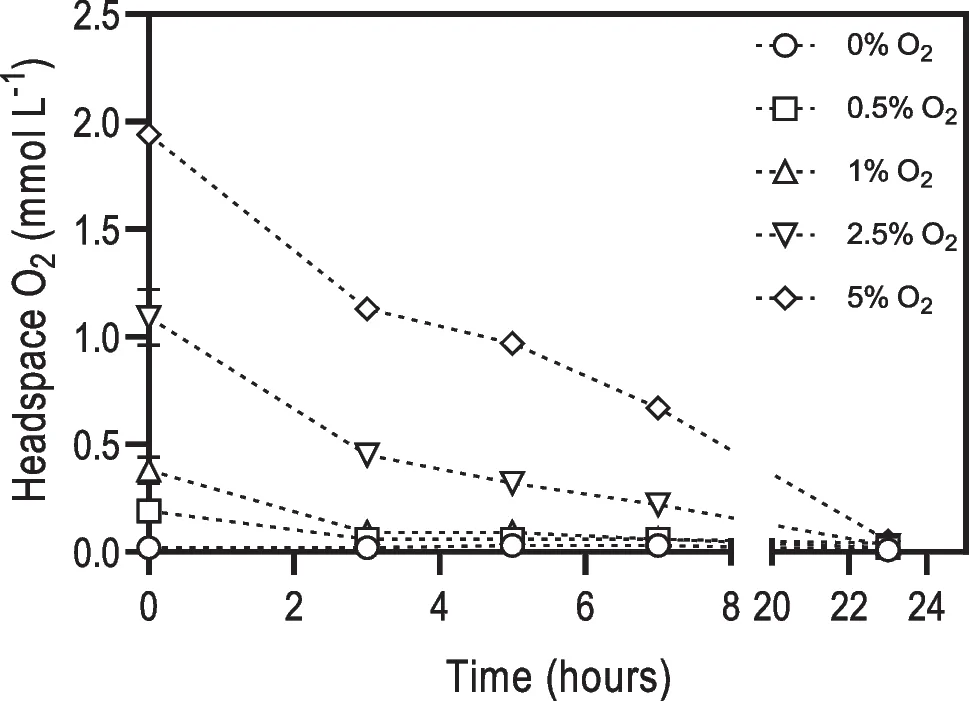
O_2_ concentration in the headspace of the blank assays during P2. Each data point represents the average of triplicates ± standard deviation

Since almost no methane was produced in the blanks, it was decided to measure oxygen concentration in the headspace over the time during P2 in these assays, to evaluate if aerobic metabolism was occurring (Fig. 2). Indeed, O_2_ was rapidly depleted in the first 3 h of incubation in the assays with 0.5% and 1% O_2_ and was reduced by 71 ± 4% and 50 ± 1% of the initial concentration in the 2.5% and 5% O_2_ conditions, respectively, after 7 h of incubation. After 24 h, only vestigial amounts of O_2_ were detected in all conditions, showing the occurrence of aerobic activity in the microbial community, even in the absence of any added substrate.

Even though P2 lasted less than 5 h in the assays with ethanol, these cultures were able to maintain their methanogenic communities active, compared to the blank assays (Fig. 1C and 1D, Table 2), albeit O_2_ exposure was significant throughout this phase. In fact, for the assays with ethanol and 5% O_2_, oxygen uptake after approximately 5 h of incubation was 58 ± 3% higher compared to the blank assays, i.e., 1.52 ± 0.06 mmol L^−1^ and 0.96 ± 0.03 mmol L^−1^, respectively.

## Discussion

Antioxidative features have been identified for both hydrogenotrophic and aceticlastic methanogens (Khademian and Imlay 2021; Lu and Imlay 2021; Lyu and Lu 2018). As such, methanogenic communities should be able to overcome oxidative stress under micro-aerobic conditions, particularly within mixed cultures. In the present study, we verified that, in the presence of O_2_, methanogenic activity was significantly reduced relatively to the control, i.e., 30–40% at 0.5% O_2_ to close to 100% inhibition at 5% O_2_ (for both acetate and H_2_/CO_2_) (Table 2). Similar results were obtained by Jasso-Chávez et al. (2015) who reported a decrease of 40% in methane production from acetate or methanol by *Methanosarcina acetivorans*, as well as a decrease in protein content of about 35–40%, when pure cultures of this archaeon were grown with 0.4–1% O_2_, relatively to control cultures grown without O_2_.

Unlike H_2_/CO_2_ and acetate, which are direct substrates for methanogenesis, ethanol is an indirect substrate that can be transformed by a wider range of microorganisms. Under methanogenic conditions, ethanol is generally oxidized by syntrophic bacteria to acetate and H_2_ (reaction 1—Table 1) (Schink 1985; Thiele and Zeikus 1988) which are further converted to methane by aceticlastic and hydrogenotrophic methanogens (reactions 2 and 3—Table 1). Ethanol oxidation to acetate is an endergonic reaction (Δ*G*°′=+9.6 kJ reaction^−1^, reaction 1—Table 1) that becomes exergonic at low hydrogen partial pressure, achieved by the activity of a hydrogenotrophic methanogenic partner (Δ*G*°′=−111.8 kJ reaction^−1^, reaction 4—Table 1). Therefore, ethanol oxidation is dependent on hydrogenotrophic methanogens, and complete ethanol conversion to methane is attained when aceticlastic methanogens are active as well, turning the overall reaction even more exergonic (Δ*G*°′=−183.8 kJ reaction^−1^, reaction 5—Table 1).

In this work, due to the low aceticlastic activity of the inoculum, the methane produced from ethanol resulted mainly from H_2_ consumption, similarly to other works that reported larger fractions of methane originating from hydrogenotrophic activity rather than from aceticlastic activity during ethanol oxidation (Liu et al. 2013; Metje and Frenzel 2005; Wu et al. 1991).

Despite the direct inhibition of methanogenic activity by O_2_ (as verified in the assays performed with H_2_/CO_2_ or acetate), ethanol conversion to methane was only slightly inhibited at O_2_ concentrations up to 1%, i.e., less than 7% inhibition of ethanol consumption rate and less than 20% inhibition of the MPR (Table 2, Table 3). At 2.5% O_2_, the rate of these two processes (ethanol consumption and methane production) was inhibited by 20 ± 1% and 36 ± 7%, respectively. Nevertheless, at 5% O_2_, inhibition of the microbial community was evident, since only 25% of the added ethanol was consumed (Table 3), and ethanol consumption rate and MPR were inhibited by 79 ± 6% and 92 ± 8%, respectively (Table 2, Table 3). These results show that the addition of ethanol resulted in a lower inhibition of hydrogenotrophic methanogens, compared to the assays with H_2_/CO_2_, and that ethanol-degrading bacteria were only marginally inhibited by O_2_ concentrations up to 2.5%. Therefore, the cultures with ethanol showed an overall resilience towards oxygen toxicity. This fact is most probably associated with the activity of facultative anaerobic bacteria and/or aerobic bacteria (FAB/AB), since the O_2_ added to the bottles was practically consumed in less than 24 h and the cultures with ethanol showed faster O_2_ consumption than the blanks (Table 4, Fig. 2). Furthermore, at 5% O_2_, almost all the ethanol consumed during P2 was converted to acetate (Table S2, Table 3), as predicted by the stoichiometry of reaction 1 (Table 1). However, the methane produced (2.4 ± 0.3 mmol L^−1^ CH_4_) was significantly lower (*p* < 0.05) than the value expected from the hydrogen potentially produced in this reaction (i.e., 3.5 ± 0.4 mmol L^−1^ CH_4_ from 14.0 ± 1.8 mmol L^−1^ H_2_, reactions 3 and 4—Table 1). Hydrogen concentration in the headspace was lower than 0.15 mmol L^−1^, showing that it was not accumulating in the bottles’ headspace. All these results taken together point to the occurrence of aerobic ethanol oxidation.

These aerobic reactions may be accomplished, for example, by acetic acid bacteria (AAB). AAB can perform aerobic ethanol oxidation to acetate that is released to the surrounding environment (Gullo et al. 2014; Saichana et al. 2015; Yamada and Yukphan 2008). This process is carried out by membrane-bound dehydrogenases that are strictly bound to the respiratory chain, and the electrons generated by the reactions are transferred by ubiquinone to O_2_, which acts as the final electron acceptor (Gullo et al. 2014; Mamlouk and Gullo 2013; Wang et al. 2015). Although AAB are considered obligate aerobes, some species can grow during alcoholic fermentation of wine (du Toit and Lambrechts 2002), and micro-aeration was shown to stimulate the growth of AAB (du Toit et al. 2006).

For each mole of ethanol oxidized to acetate by AAB, one mole of O_2_ is required. In the present work, considering the O_2_ uptake measured (Table 4), the maximum ethanol oxidation by AAB would be approximately 0.9 mmol L^−1^ and 1.5 mmol L^−1^ for 2.5% and 5% O_2_, which does not justify the significant differences (*p* < 0.05) observed in ethanol consumption and methane production at these two conditions. Therefore, the presence of ethanol provided an alternative aerobic pathway that enhanced the O_2_ removal from the media, allowing the methanogenic community to maintain its activity, but this phenomenon was not enough to circumvent the inhibition caused by the higher oxygen concentration tested (2.5% and 5% O_2_). Still, the inhibitory effects were substantially minimized.

Although AAB are also capable of acetate oxidation once other carbon sources are depleted (Gullo et al. 2014; Saeki et al. 1997; Sakurai et al. 2012), this was not observed in the assays that received ethanol, since acetate uptake was not observed throughout P2. Also, in the assays with acetate, no significant acetate uptake was observed in P2 at 5% O_2_, and at 2.5% O_2_, the acetate was mostly converted to methane. Acetate oxidation by AAB generally occurs after a prolonged lag phase (~100 h), and a steady aeration rate is generally applied (Saeki et al. 1997), which was not provided in our experiments, thus it is unlikely that acetate oxidation was a viable metabolic pathway in the conditions set for the assays with ethanol or acetate.

In conclusion, hydrogenotrophic and aceticlastic methanogens were inhibited by oxygen, presenting significantly lower MPR than the controls already at 0.5% O_2_, and reaching close to 100% inhibition at 5% O_2_. The cultures with ethanol showed an overall resilience towards oxygen toxicity up to 2.5% O_2_, with significant inhibitory effects being observed for oxygen concentrations higher than that. Therefore, the presence of ethanol favored the occurrence of an alternative aerobic pathway that enhanced oxygen removal, allowing the microbial community to maintain its activity at oxygen concentrations up to 2.5%.

At industrial applications, oxygen contamination should be avoided as much as possible, to minimize the inhibitory effect of oxygen on methanogens and other strict anaerobes in anaerobic digestion processes, as well as unwanted aerobic substrate conversion that may limit the methane yield from a given substrate. However, because strict anaerobic conditions are typically not enforced for practical or financial reasons, trace amounts of oxygen are frequently present in full-scale anaerobic digesters. This work shows that the activity of FAB/AB provides a shielding effect towards syntrophic methanogenic communities, limiting the inhibitory effect of oxygen, and thus, cost-benefit calculations in industrial applications should include the protective effect of these bacteria on anaerobic processes. The present work uses ethanol as a syntrophic substrate, but other fatty acids, such as propionate or butyrate, are important intermediates in anaerobic digestion processes, whose degradation also relies on syntrophic relationships. The effect of micro-aeration on the degradation of these substrates still has to be investigated. Considering the important role of syntrophy in the breakdown of organic compounds in anaerobic digestion, this work brings important insights on the toxicity of oxygen and on the role of FAB/AB in preventing, to a certain extent, the inhibitory effect of oxygen contamination.

## Supplementary information

ESM 1 (PDF 539 kb)

## Data Availability

The data that supports the findings of this study are available in the supplementary material of this article.
