# Supplementary material for: Effect of micro-aeration on syntrophic and methanogenic activity in anaerobic sludge
Source: Appl Microbiol Biotechnol. 2024 Feb 2;108(1):192. doi: 10.1007/s00253-023-12969-4 (PMC10837232; doi:10.1007/s00253-023-12969-4)
Supplement: Supplementary file 1 — (PDF 539 kb) [file 253_2023_12969_MOESM1_ESM.pdf]

Supplementary Material

Effect of micro-aeration on syntrophic and methanogenic activity in anaerobic sludge

Bruno P. Morais[1,2¥§], Carla P. Magalhães[1,2§], Gilberto Martins[1,2], Maria Alcina Pereira[1,2], Ana J. Cavaleiro[1,2#]

[1] CEB - Centre of Biological Engineering, University of Minho, Braga, Portugal.

[2] LABBELS –Associate Laboratory, Braga/Guimarães, Portugal.

[§] These authors equally contributed to this work.

[#] Corresponding author: A.J. Cavaleiro, acavaleiro@deb.uminho.pt

[¥] Current address: CICECO, Aveiro Institute of Materials, Universidade de Aveiro, Portugal

List of Figures

Figure S1. Effect of different O_2 concentrations on cumulative methane production from H_2/CO_2, in P1 (●) and P2, (○): 0 % O_2 (A), 0.5 % O_2 (B), 1 % O_2 (C), 2.5 % O_2 (D) and 5 % O_2 (E). Dashed lines show the data points used to calculate the MPR in P1 (black dashed lines) and P2 (grey dashed lines). (↓) indicates the moment of air addition and (↑) indicates H_2/CO_2 replenishment. Each data point represents the average of triplicates ± standard deviation. 4

Figure S2. Effect of different O_2 concentrations on cumulative methane production from acetate, in P1 (●) and P2, (○): 0 % O_2 (A), 0.5 % O_2 (B), 1 % O_2 (C), 2.5 % O_2 (D) and 5 % O_2 (E). Dashed lines show the data points used to calculate the MPR in P1 (black dashed lines) and P2 (grey dashed lines). (↓) indicates the moment of air addition and (↑) indicates acetate replenishment. Each data point represents the average of triplicates ± standard deviation. 5

Figure S3. Effect of different O_2 concentrations on cumulative methane production from ethanol, in P1 (●) and P2, (○): 0 % O_2 (A), 0.5 % O_2 (B), 1 % O_2 (C), 2.5 % O_2 (D) and 5 % O_2 (E). Dashed lines show the data points used to calculate the MPR in P1 (black dashed lines) and P2 (grey dashed lines). (↓) indicates the moment of air addition and (↑) indicates ethanol replenishment. Each data point represents the average of triplicates ± standard deviation. 6

Figure S4. Ethanol (●) and acetate (○) concentrations during P2 in the assays with ethanol and increasing O_2 concentrations: 0 % O_2 (A), 0.5 % O_2 (B), 1 % O_2 (C), 2.5 % O_2 (D) and 5 % O_2 (E). Dashed lines show the data points used to calculate the ethanol uptake ratio (black dashed lines) and acetate production rate (grey dashed lines). Each data point represents the average of triplicates ± standard deviation. 7

List of Tables

Table S1. H_2 and acetate concentrations at the beginning of P1 and P2, and total consumption (average of triplicates ± standard deviation). 8

Table S2. Ethanol and acetate concentrations measured at the beginning (t_0) and end (t_f) of P1 and P2, at increasing O_2 concentrations, in the assays with ethanol. Values are the average of triplicates ± standard deviation. 9

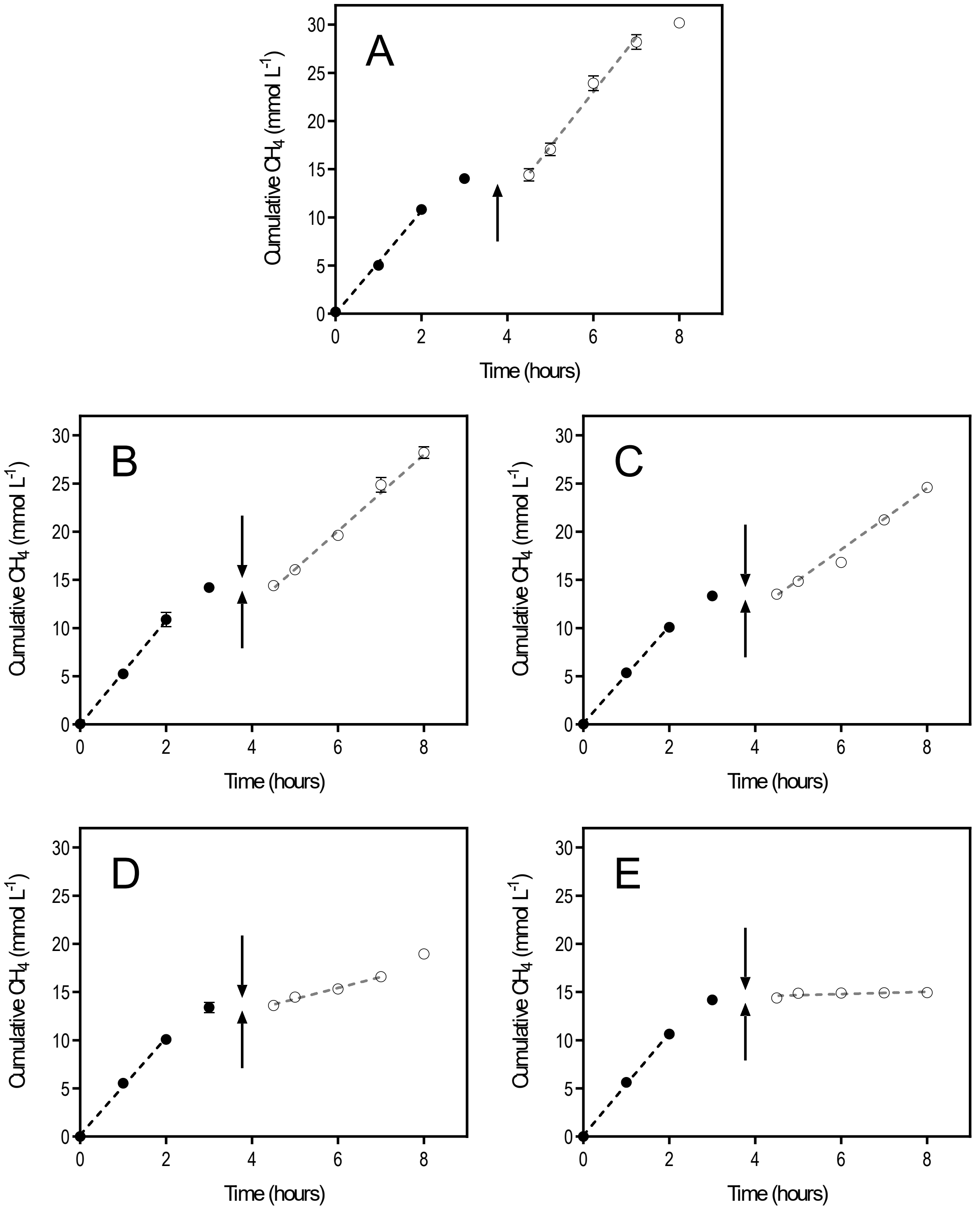

Figure S1. Effect of different O_2 concentrations on cumulative methane production from H_2/CO_2, in P1 (●) and P2, (○): 0 % O_2 (A), 0.5 % O_2 (B), 1 % O_2 (C), 2.5 % O_2 (D) and 5 % O_2 (E). Dashed lines show the data points used to calculate the MPR in P1 (black dashed lines) and P2 (grey dashed lines). (↓) indicates the moment of air addition and (↑) indicates H_2/CO_2 replenishment. Each data point represents the average of triplicates ± standard deviation.

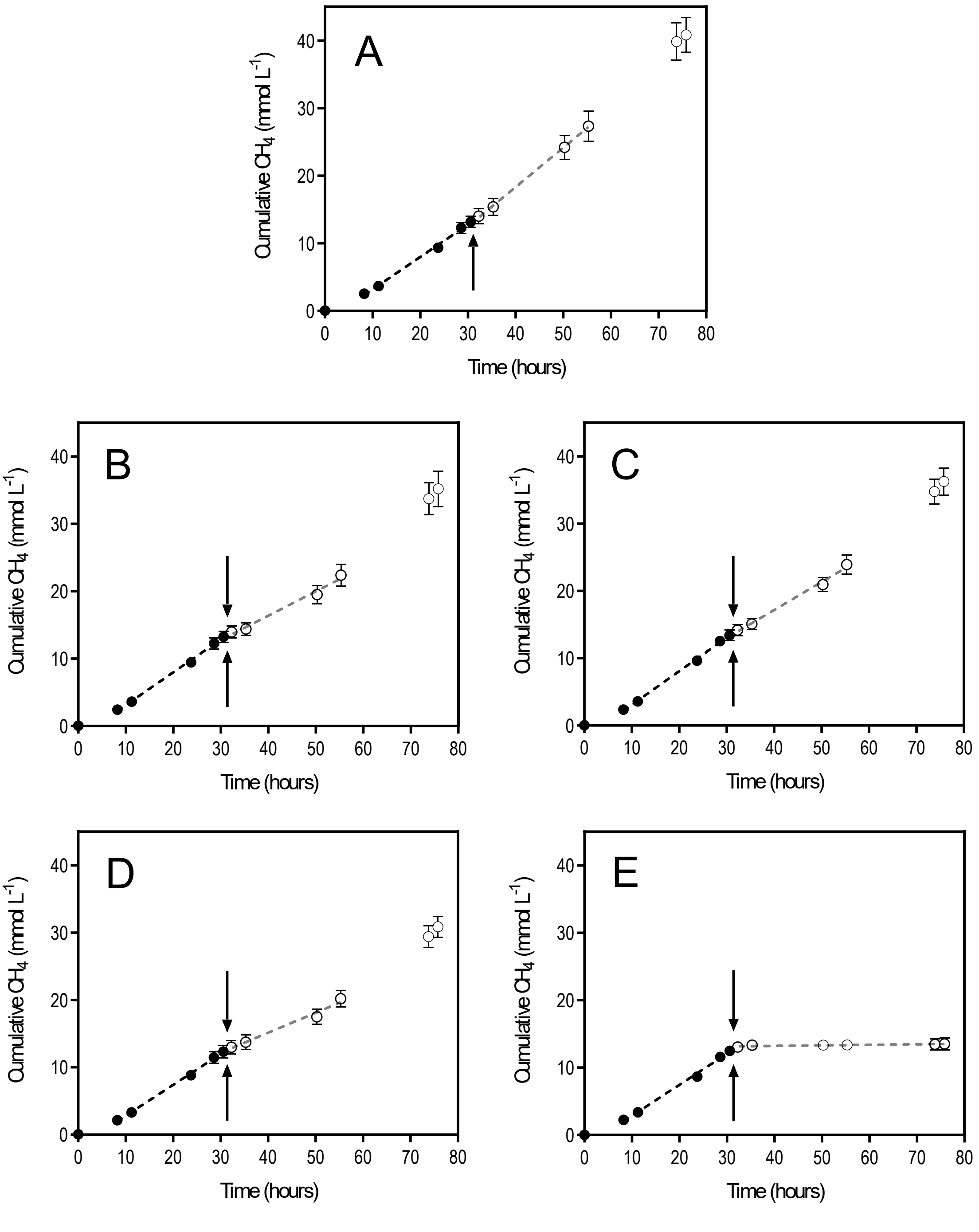

Figure S2. Effect of different O_2 concentrations on cumulative methane production from acetate, in P1 (●) and P2, (○): 0 % O_2 (A), 0.5 % O_2 (B), 1 % O_2 (C), 2.5 % O_2 (D) and 5 % O_2 (E). Dashed lines show the data points used to calculate the MPR in P1 (black dashed lines) and P2 (grey dashed lines). (↓) indicates the moment of air addition and (↑) indicates acetate replenishment. Each data point represents the average of triplicates ± standard deviation.

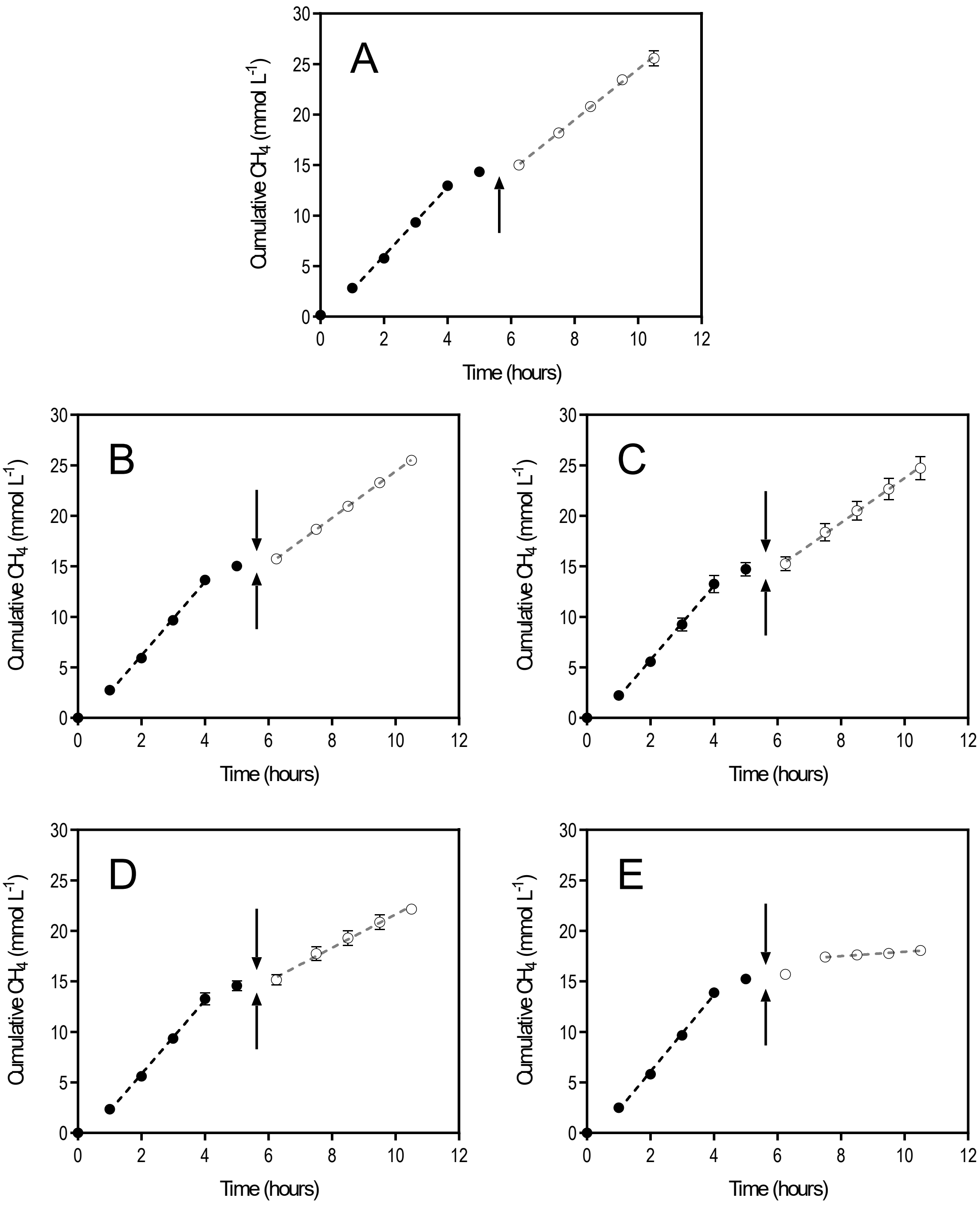

Figure S3. Effect of different O_2 concentrations on cumulative methane production from ethanol, in P1 (●) and P2, (○): 0 % O_2 (A), 0.5 % O_2 (B), 1 % O_2 (C), 2.5 % O_2 (D) and 5 % O_2 (E). Dashed lines show the data points used to calculate the MPR in P1 (black dashed lines) and P2 (grey dashed lines). (↓) indicates the moment of air addition and (↑) indicates ethanol replenishment. Each data point represents the average of triplicates ± standard deviation.

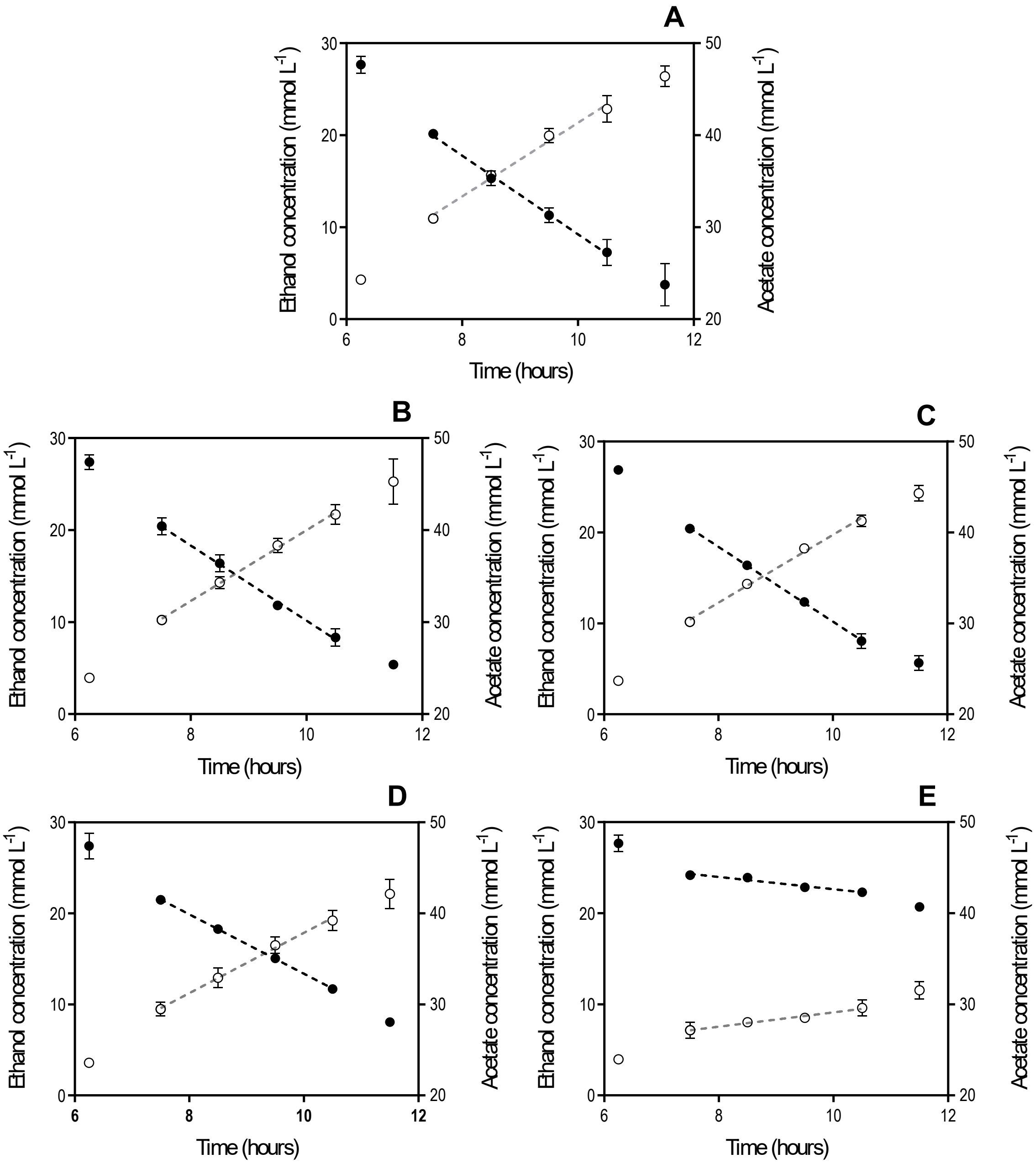

Figure S4. Ethanol (●) and acetate (○) concentrations during P2 in the assays with ethanol and increasing O_2 concentrations: 0 % O_2 (A), 0.5 % O_2 (B), 1 % O_2 (C), 2.5 % O_2 (D) and 5 % O_2 (E). Dashed lines show the data points used to calculate the ethanol uptake ratio (black dashed lines) and acetate production rate (grey dashed lines). Each data point represents the average of triplicates ± standard deviation.

Table S1. H_2 and acetate concentrations at the beginning of P1 and P2, and total consumption (average of triplicates ± standard deviation).

Substrate	P1		P2		
	**Initial concentration (mmol L^{-1}) [(a)]**	**Total consumption (mmol L^{-1})**	**O_2 (%)**	**Initial concentration (mmol L^{-1}) [(a)]**	**Total consumption (mmol L^{-1})**
H_2/CO_2	55.6 ± 0.6	48.6 ± 2.2	0	53.2 ± 1.2	49.3 ± 1.4
	53.5 ± 0.5	48.2 ± 1.5	0.5	51.6 ± 0.5	43.5 ± 1.5
	53.4 ± 0.3	44.9 ± 0.7	1.0	48.5 ± 2.3	32.8 ± 2.6
	53.2 ± 0.3	46.0 ± 1.1	2.5	46.1 ± 0.6	12.9 ± 1.1
	53.5 ± 0.5	48.3 ± 3.6	5.0	42.2 ± 0.2	0.0 ± 0.0
Acetate	24.3 ± 0.6	12.1 ± 1.5	0	25.4 ± 2.1	24.0 ± 2.6
	25.2 ± 0.3	12.6 ± 0.7	0.5	25.9 ± 0.5	17.6 ± 1.5
	23.7 ± 2.0	11.4 ± 2.0	1.0	25.2 ± 0.6	19.3 ± 0.7
	24.0 ± 0.8	10.6 ± 1.2	1.5	25.9 ± 0.7	16.0 ± 1.3
	24.7 ± 0.9	11.7 ± 1.3	2.0	26.6 ± 1.1	0.4 ± 1.9

[(a)] H_2 concentration was measured in H_2/CO_2 assays.

Table S2. Ethanol and acetate concentrations measured at the beginning (t_0) and end (t_f) of P1 and P2, at increasing O_2 concentrations, in the assays with ethanol. Values are the average of triplicates ± standard deviation.

P1				P2				
**[Eth] t_0 (mmol L^{-1})**	**[Eth] t_f (mmol L^{-1})**	**[Ac] t_0 (mmol L^{-1})**	**[Ac] t_f (mmol L^{-1})**	**O_2 (%)**	**[Eth] t_0 (mmol L^{-1})**	**[Eth] t_f (mmol L^{-1})**	**[Ac] t_0 (mmol L^{-1})**	**[Ac] t_f (mmol L^{-1})**
26.6 ± 0.0	0.0 ± 0.0	0.1 ± 0.0	24.9 ± 0.2	0	27.7 ± 0.8	3.8 ± 1.9	24.3 ± 0.3	46.4 ± 0.9
26.1 ± 0.4	0.0 ± 0.0	0.1 ± 0.0	24.6 ± 0.4	0.5	27.4 ± 0.7	5.4 ± 0.4	23.9 ± 0.6	45.3 ± 2.0
26.1 ± 0.4	0.0 ± 0.0	0.1 ± 0.0	24.4 ± 0.1	1.0	26.9 ± 0.4	5.6 ± 0.7	23.7 ± 0.1	44.3 ± 0.7
25.7 ± 0.6	0.0 ± 0.0	0.1 ± 0.0	24.2 ± 0.5	2.5	27.4 ± 1.1	7.2 ± 1.2	23.6 ± 0.6	42.1 ± 1.1
26.2 ± 0.3	0.0 ± 0.0	0.1 ± 0.0	24.3 ± 0.2	5.0	27.7 ± 0.8	20.7 ± 0.4	24.0 ± 0.1	31.6 ± 0.8

Eth (Ethanol), Ac (Acetate)
